# Adenomatoid tumors of the uterus: magnetic resonance imaging features with clinical and histopathologic correlation

**DOI:** 10.3389/fonc.2026.1942362

**Published:** 2026-09-08

**Authors:** Changhuan Chen, Yudian Huang, Ping Chen, Caixia Zheng, Chengsheng Wang, Mingsi Yang

**Affiliations:** 1 Department of Radiology, Fuzhou First General Hospital Affiliated with Fujian Medical University, Fuzhou, China; 2 Department of Pathology, Fuzhou First General Hospital Affiliated with Fujian Medical University, Fuzhou, China; 3 Department of Radiology, Clinical Oncology School of Fujian Medical University, Fujian Cancer Hospital, Fuzhou, China

**Keywords:** adenomatoid tumor, diffusion weighted imaging, magnetic resonance imaging, pathology, uterus

## Abstract

**Purpose:**

Adenomatoid tumor (AT) of the uterus is a rare benign neoplasm of mesothelial origin. To date, few studies have reported the magnetic resonance imaging (MRI) findings and clinicopathologic features of uterine ATs. This study aimed to summarize the MRI features of uterine ATs and relate them to their clinicopathologic findings, with the goal of improving the accuracy of preoperative diagnosis.

**Methods:**

Sixteen surgically resected, histopathologically confirmed uterine ATs were retrospectively reviewed. Clinical data, imaging characteristics, and histopathologic findings were systematically analyzed.

**Results:**

The 16 patients had a median age of 51 years. ATs coexisted with uterine fibroids in 8 of the 16 patients. Among the 16 patients, 15 each had a single AT, and one patient had two synchronous ATs, for a total of 17 lesions. Seven lesions were intramural, and 10 were subserosal. All 17 lesions were oval, with a maximum diameter of 9–65 mm; four had ill-defined margins and 13 had well-defined margins; no identifiable capsule was seen. Thirteen lesions were solid, two were cystic-solid, and two were cystic. The solid components were isointense on T1-weighted imaging (T1WI) and predominantly hypointense on T2-weighted imaging (T2WI). Small foci of cystic degeneration were present in five lesions, and four exhibited a “target sign” (central hypointensity with a peripheral annular hyperintense rim) on T2WI. Solid components demonstrated mild diffusion restriction in most lesions. The solid components demonstrated moderate-to-marked enhancement, comparable to or slightly less than that of the adjacent myometrium. The cystic components were hypointense on T1WI and hyperintense on T2WI without enhancement. Two cystic lesions with septa, both showed peripheral enhancement and progressive enhancement of the internal septa. Imaging findings correlated closely with histopathologic findings. The tumors consisted of gland-like spaces of varying sizes interspersed with smooth muscle and fibrous tissue. Differences in the relative proportions of these components accounted for the diverse imaging appearances.

**Conclusions:**

Uterine ATs predominantly present as solid masses without an identifiable capsule. Small foci of cystic degeneration or a “target sign” on T2WI, observed in a subset of cases, may represent nonspecific imaging findings of uterine ATs. However, definitive diagnosis still requires histopathologic examination.

## 1 Introduction

Adenomatoid tumor (AT) is a rare benign tumor of mesothelial origin (1). The pathogenesis of AT may involve mutations in the *TRAF7* gene, which lead to abnormal activation of the NF-κB pathway and subsequent tumor growth (2). ATs typically occur in the genital tract, with the uterus being one of the most common sites. Because uterine ATs lack specific clinical manifestations and laboratory markers, they are often incidentally discovered during routine histopathological examination following myomectomy or hysterectomy (3). Surgery is the mainstay of treatment for uterine ATs and includes local excision or hysterectomy. However, because ATs are tightly adherent to the surrounding uterine myometrium or protrude subserosally, imaging analyses are needed to confirm the tumor diagnosis, assess lesion extent, and determine its anatomical relationship between the tumor and adjacent organs (4). Furthermore, previous studies have reported that the magnetic resonance imaging (MRI) findings of ATs closely resemble those of uterine leiomyomas, making them prone to misdiagnosis or missed diagnosis (4, 5). Additionally, cystic changes may occur within the tumor, and some lesions may even appear cystic, which is another major cause of misdiagnosis (6–8). Notably, uterine leiomyomas can often be medically treated, whereas the efficacy of such medicines for AT remains unclear; if an AT is misdiagnosed as a leiomyoma based solely on imaging findings, the patient may undergo unnecessary medical intervention. Therefore, accurate preoperative diagnosis and evaluation are clinically significant for avoiding overtreatment and guiding optimal treatment planning for patients.

At present, reports on the imaging features of uterine ATs remain limited, and most of them are case reports (4, 5, 9, 10). By analyzing MRI data from 16 patients with uterine ATs and correlating them with their clinical and pathological findings, this study aims to gain a deeper understanding of the imaging characteristics of this rare disease, thereby improving the accuracy of preoperative diagnosis.

## 2 Materials and methods

### 2.1 Patients

This retrospective study was approved by the Ethics Committee of our institution (No. 202210028), and the requirement for informed consent was waived. We searched the pathology database to identify all patients with a histopathologic diagnosis of uterine ATs between June 2016 and January 2026, for a total of 71 patients. We then used the Picture Archiving and Communication System (PACS) to determine which of these 71 patients had undergone contrast-enhanced pelvic MRI before surgery; 33 patients met this criterion, while the remaining 38 patients, who lacked preoperative MRI, were excluded. These 33 patients were further evaluated against the following exclusion criteria: (1) coexisting masses that precluded accurate localization of the AT on MRI, making radiologic-pathologic correlation unreliable (n = 9); and (2) poor MRI image quality or a tumor diameter <5 mm that was not clearly identifiable on MRI, precluding accurate evaluation (n = 6), leaving 18 patients for further analysis. Electronic medical records were subsequently reviewed, and patients were excluded if they had (1) a history of prior treatment, including medical therapy, interventional procedures, or surgery (n = 1), or (2) incomplete clinical data (n = 1). After applying these exclusion criteria, a final cohort of 16 patients was retrospectively included. Surgical records and clinical data were subsequently retrieved from the electronic medical records. Collected information included demographic characteristics, clinical manifestations, and surgical indications.

### 2.2 MR imaging protocol

All 16 patients underwent contrast-enhanced pelvic MRI before surgery. Seven patients were examined using a 1.5-T MRI scanner (Avanto, Siemens). The imaging protocol included axial T1-weighted gradient-echo (GRE) imaging (repetition time, TR, 185 ms; echo time TE, 7.2 ms); coronal T2-weighted true fast imaging with steady state precession (True FISP) imaging (TR, 3.88 ms; TE, 1.62 ms); axial and sagittal T2-weighted turbo spin-echo (TSE) imaging (TR, 3390–5310 ms; TE, 93–125 ms); and axial diffusion-weighted single-shot echo-planar imaging (DWI-SE EPI) (TR, 2500 ms; TE, 77 ms; b values, 50 and 800 s/mm²). The field of view (FOV) ranged from 230 × 230 mm to 500 × 500 mm, with a slice thickness of 4–5 mm. For contrast-enhanced imaging, gadopentetate dimeglumine (Gd-DTPA) was administered intravenously through the cubital vein at a dose of 0.2 mL/kg and an injection rate of 2 mL/s, followed by axial, coronal, and sagittal fat-suppressed spoiled T1-weighted GRE imaging. Nine patients were examined using a 3.0-T MRI scanner (Ingenia CX, Philips). The imaging protocol included axial, coronal, and sagittal T2-weighted TSE imaging (TR, 2702 ms; TE, 100 ms); axial T1-weighted TSE imaging (TR, 543 ms; TE, 20 ms); and axial DWI SE EPI (TR, 5352 ms; TE, 75 ms; b values, 50 and 800 s/mm²). The FOV ranged from 200 × 200 mm to 500 × 500 mm, with a slice thickness of 4–5 mm. Contrast-enhanced imaging was performed after intravenous administration of Gd-DTPA (0.2 mL/kg at 2 mL/s), followed by axial, coronal, and sagittal T1-weighted modified Dixon imaging.

### 2.3 Imaging analysis and measurement methods

Two radiologists, each with more than 10 years of experience in pelvic imaging, independently and blindly reviewed all MRI examinations. The evaluated imaging features included tumor number, location, size, shape, margin, internal architecture, composition (cystic, cystic-solid, or solid), signal characteristics, and enhancement pattern. The imaging findings were subsequently correlated with the corresponding pathological findings. Any discrepancies were resolved by consensus. Tumor location was classified according to its relationship with the uterine wall as subserosal, intramural, or submucosal. Tumor size was defined as the maximum diameter measured on T2WI. Tumor shape was categorized as round/oval or irregular, and margins were classified as well-defined or ill-defined. Signal intensity was assessed relative to that of the normal uterine myometrium. Tumor composition was classified as cystic (>80% cystic component), solid (>80% solid component), or cystic-solid (20%–80% cystic component). When image quality permitted, diffusion-weighted imaging (DWI) was carefully evaluated, and apparent diffusion coefficient (ADC) values were measured for the solid components. Contrast enhancement was visually graded into three categories: mild enhancement, similar to adjacent skeletal muscle; moderate enhancement, greater than skeletal muscle but less than the uterine myometrium; and marked enhancement, approaching that of the adjacent myometrium.

All measurements were performed by a single radiologist with more than 10 years of experience in diagnostic imaging, who was blinded to the clinical and pathological findings. The measurement protocol for tumor size and ADC values was as follows. First, the raw images were uploaded to the respective workstations (Syngo, Siemens, Germany; IntelliSpace Portal, Philips, The Netherlands), which automatically generated the ADC maps. Using each workstation and referring to the signal characteristics on T1WI, T2WI, and contrast-enhanced images, the tumor’s longest diameter was measured on T2WI. For each lesion, the measurement was performed three times, and the mean diameter was used as the final tumor size. Subsequently, on the corresponding ADC map, the slice demonstrating the largest solid tumor component was selected for region of interest (ROI) placement. The ROI was manually placed along the inner margin of the solid tumor, encompassing as much of the solid component as possible while avoiding areas of cystic change, necrosis, and hemorrhage; the ROI boundary was positioned slightly within the solid tumor margin to minimize the partial volume effect. ROI placement was repeated three times for each lesion, and the mean of the three resulting ADC values was recorded as the final measurement.

### 2.4 Histopathological analyses

All 16 surgical specimens underwent routine fixation, paraffin embedding, sectioning, and hematoxylin and eosin (H&E) staining. Gross and microscopic findings were reviewed by a gynecologic pathologist with more than 10 years of experience. Histopathologic findings were subsequently correlated with the corresponding MRI findings. A radiologist and a pathologist jointly performed the imaging-pathologic correlation. They compared the location, shape, and signal characteristics of lesions on MRI with the corresponding histopathologic sections on a case-by-case basis. For quantifiable parameters such as tumor size, Pearson correlation analysis was used to assess the relationship between MRI-derived measurements and quantitative histopathologic indices.

## 3 Results

### 3.1 Clinical findings

The patients ranged in age from 27 to 63 years, with a median age of 51 years. Clinically, eight patients were asymptomatic. Five patients presented with dull lower abdominal pain accompanied by menstrual abnormalities, including irregular cycles, increased menstrual flow, or prolonged menstrual duration. The remaining three patients presented with abnormal vaginal bleeding. The past medical history of the 16 patients was varied, mainly including no significant medical history, tubal ligation, and cesarean section.

### 3.2 MRI findings

**Figure 1:**
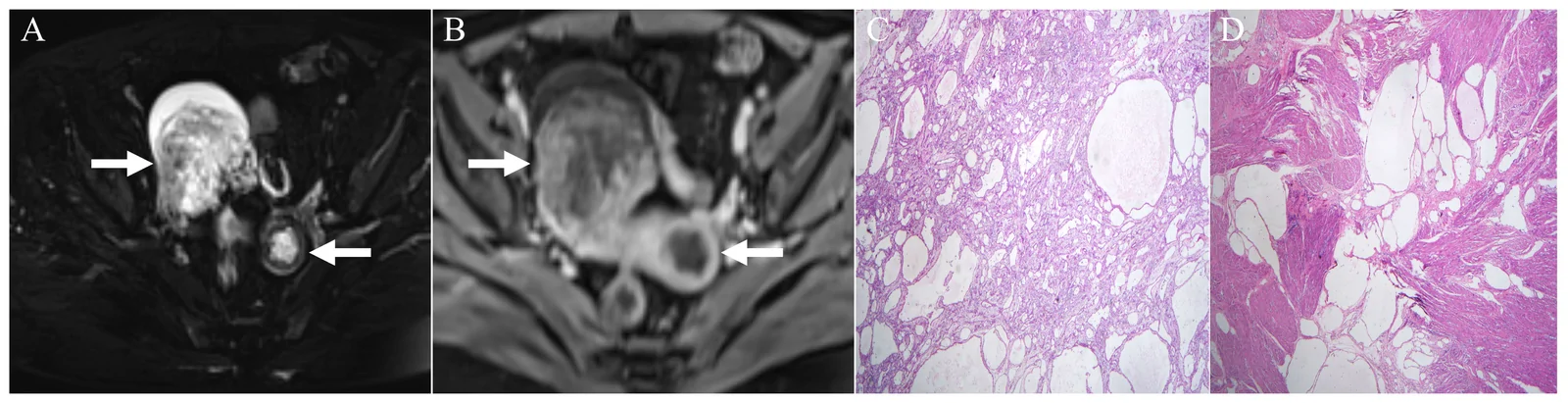
A 49-year-old woman with AT of the uterus. **(A)** Axial T2-weighted imaging shows a subserosal mass in each uterine horn, with the right-sided lesion appearing cystic-solid and the left-sided lesion appearing cystic (arrows). **(B)** Contrast-enhanced axial T1-weighted imaging shows heterogeneous enhancement in the right lesion, while rim enhancement in the left lesion. **(C, D)** Histopathologic specimen shows that both masses consist of glandular spaces, smooth muscle bundles, and fibrous tissue, consistent with AT; however, the proportions of these components differ between the two lesions (H&E ×25).

**Figure 2:**
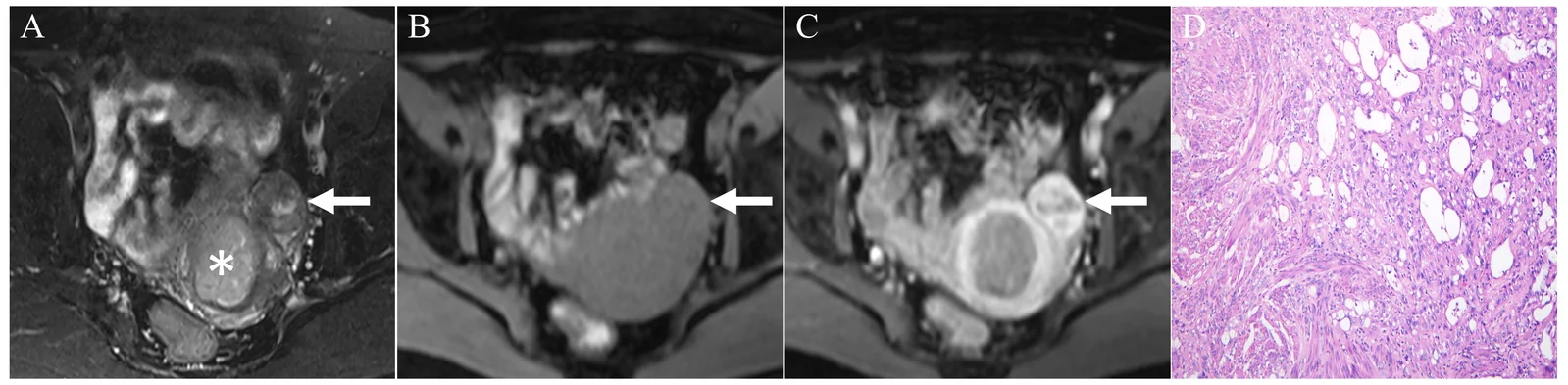
A 56-year-old woman with solid AT of the uterus. **(A)** Axial T2-weighted imaging shows a hypointense mass with small foci of cystic degeneration (arrow) and abnormal endometrial thickening (asterisk, endometrial carcinoma). **(B)** Axial T1-weighted imaging shows the tumor with signal intensity isointense to the myometrium. **(C)** Contrast-enhanced axial T1-weighted imaging shows marked heterogeneous enhancement of the tumor. **(D)** Histopathologic specimen shows the tumor is composed of variably sized glandular spaces, smooth muscle bundles, and fibrous tissue arranged in a random pattern, consistent with AT (H&E ×100).

**Figure 3:**
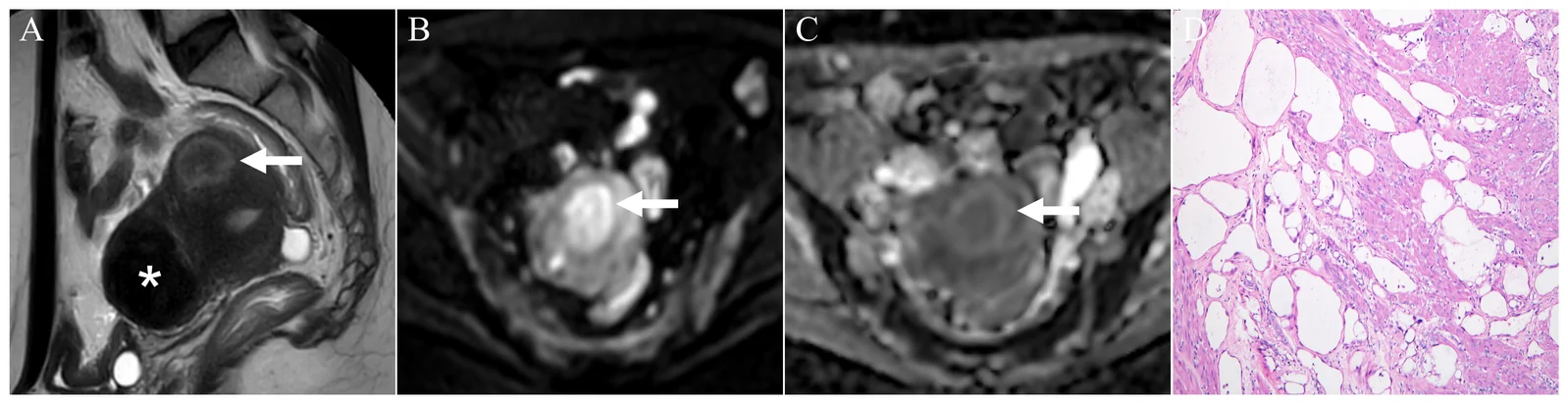
A 43-year-old woman with solid AT of the uterus. **(A)** Sagittal T2-weighted imaging shows a “target sign” within the mass, characterized by central hypointensity with a peripheral annular hyperintense rim (arrow), which differs from the appearance of the other intramural uterine mass (asterisk, uterine fibroid). **(B)** The tumor appears slightly heterogeneously hyperintense on axial DWI imaging. **(C)** The tumor also shows a “target sign” on the ADC map, with a relatively high ADC value (peripheral hyperintensity, mean 1.55×10–3 mm2/s; central hypointensity, mean 1.27×10–3 mm2/s). **(D)** Histopathologic specimen shows glandular spaces of variable size lined by flattened or low columnar epithelial cells, arranged in a concentric pattern and admixed with smooth muscle bundles and fibrous tissue (H&E ×100).

**Figure 4:**
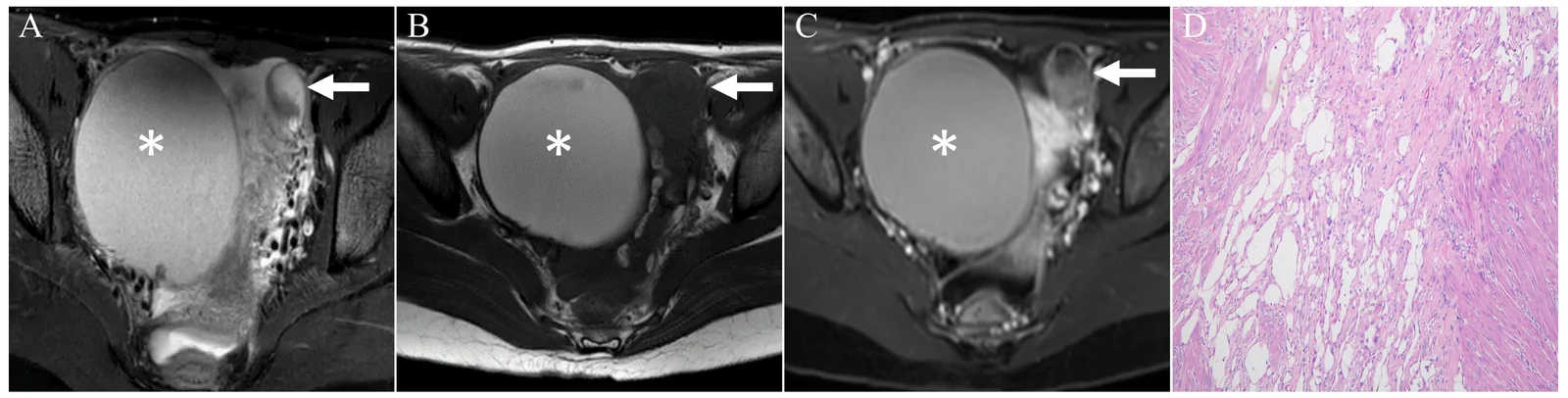
A 27-year-old woman with cystic-solid AT of the uterus. **(A)** Axial T2-weighted imaging shows a cystic-solid mass with well-defined margin in the subserosal space of the left uterine horn, measuring 2.5 cm in diameter (arrow). A large cystic tumor in the right ovary is also observed (asterisk, serous cystadenoma). **(B)** Axial T1-weighted imaging shows the tumor with isointense signal intensity. **(C)** Contrast-enhanced axial T1-weighted imaging shows moderate enhancement of the solid component. **(D)** Histopathologic specimen shows some of the dilated glandular spaces arranged eccentrically within the tumor (H&E ×100).

**Figure 5:**
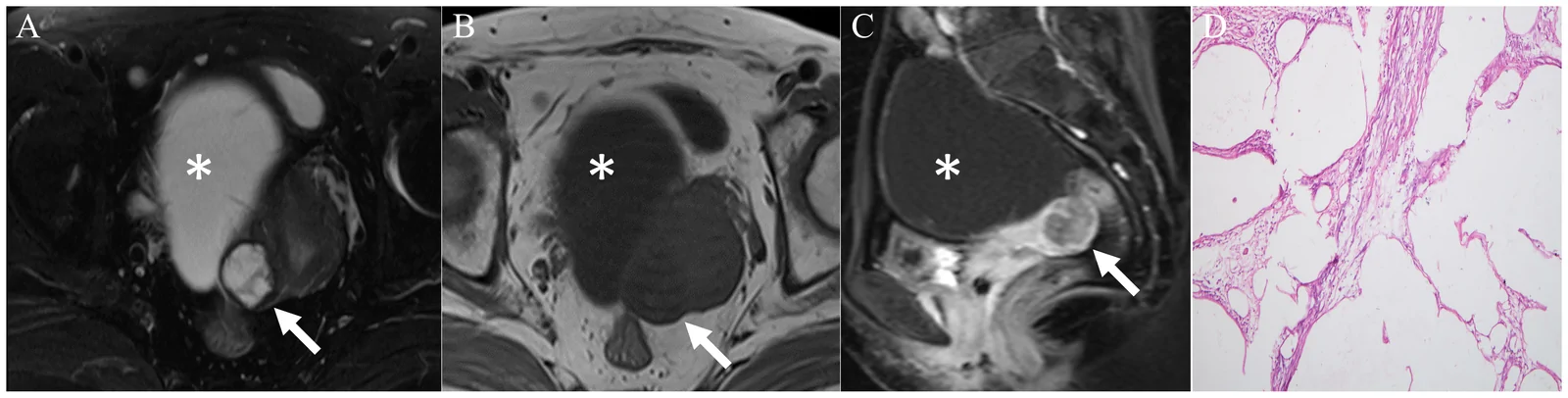
A 54-year-old woman with cystic AT of the uterus. **(A)** Axial T2-weighted imaging shows a hyperintense mass with a few internal septa (arrow). A large cystic tumor in the right ovary is also observed (asterisk, mucinous cystadenoma). **(B)** Axial T1-weighted imaging shows the tumor with slightly low signal intensity. **(C)** Contrast-enhanced sagittal T1-weighted imaging shows rim enhancement, with the internal septation demonstrating progressive enhancement. **(D)** Histopathologic specimen shows glandular spaces fused with one another, interspersed with fibrous tissue and bundles of smooth muscle (H&E ×100).

(1) Number of tumors: A total of 17 lesions were identified in 16 patients. One patient had two lesions (**Figure 1**). (2) Location: Of the 17 lesions, ten were subserosal and the remaining seven were intramural. Among the subserosal lesions, six involved the uterine horns, including four in the left horn and two in the right horn. (3) Shape and size: All lesions were oval. The maximum diameter measured on MRI ranged from 9 to 65 mm. (4) Margins: Four lesions had ill-defined margins, whereas 13 had well-defined margins. No definite capsule-like structure was observed. (5) Internal signal characteristics and composition: Of the 17 lesions, 13 were solid (**Figures 2**, **3**), two were cystic-solid (**Figure 4**), and two were cystic (**Figure 5**). Compared with the uterine myometrium, the solid components exhibited isointense signal on T1WI and hypointense signal on T2WI. The cystic components appeared hypointense on T1WI and hyperintense on T2WI. Among the 13 solid tumors, five showed small foci of cystic degeneration, and four demonstrated low central signal intensity surrounded by a slightly hyperintense peripheral ring, resembling a “target sign.” In these 15 lesions with solid components, the solid component of 11 lesions demonstrated hyperintensity on diffusion-weighted imaging (DWI; b = 800 s/mm²) with correspondingly mild hypointensity on apparent diffusion coefficient (ADC) maps. Among the four solid lesions with a “target sign” on T2WI, the peripheral rim of each lesion shows mild hyperintensity on both DWI and ADC maps. The solid components of the remaining lesions exhibited mild hypointensity on both DWI and ADC maps. No evidence of fat or hemorrhage was identified in any lesion. Contrast-enhanced MRI demonstrated moderate to marked enhancement of the solid tumor components, with enhancement similar to or slightly weaker than that of the uterine myometrium. The cystic components showed no enhancement. The two cystic tumors exhibited rim enhancement, with internal septa demonstrating progressive enhancement. (6) Associated lesions: Eight patients also had uterine fibroids, four had ovarian cystadenomas, one had endometrial cancer, one had cervical cancer, one had cervical intraepithelial neoplasia (CIN 3), one had ovarian teratoma, and one had rectal cancer. Some patients had two or more of these associated lesions.

### 3.3 Histopathological findings

All 16 patients underwent surgical resection. Gross examination: Seventeen lesions were identified, including 13 solid, two cystic-solid, and two cystic lesions. Ten lesions were subserosal, whereas the remaining seven were intramural. Six of the subserosal lesions were located at the uterine horns, including four in the left horn and two in the right horn. Tumor diameters ranged from 1.0 to 6.9 cm. The tumors were relatively soft. On the cut surface, they appeared grayish-yellow, lacked an obvious capsule, and showed no distinct boundary with the surrounding tissues. Microscopic examination: All 17 lesions exhibited essentially identical histologic features, consisting of glandular spaces of variable sizes and shapes. These spaces were lined by flattened or low columnar epithelial cells. The tumor cells were generally uniform, without cytologic atypia or mitotic figures, and had vacuolated cytoplasm. The stroma consisted of loose or dense fibrous tissue or fibrous tissue interspersed with smooth muscle bundles. Postoperative pathological examination confirmed uterine ATs in all 17 lesions. Concurrent uterine pathology was identified in a subset of patients, including uterine fibroids (n=8), cervical cancer (n=1), cervical intraepithelial neoplasia (CIN 3) (n=1), and endometrial cancer (n=1). In addition, pathological findings outside the uterus were observed, comprising ovarian cystadenoma (n=4), ovarian teratoma (n=1), and rectal cancer (n=1). Some patients had more than one of these concurrent pathologies. Immunohistochemical analysis showed that tumor cells in all cases were positive for CK, CR, and D2–40 and negative for CK5/6 and CD34.

## 4 Discussion

ATs were first described by Golden and Ash in 1945 (11). ATs originate from mesothelial tissue and typically occur in the genital tract of both men and women. In women, the uterus and fallopian tubes are the primary sites of involvement, whereas in men, these tumors are most commonly found in the epididymis (1). Rarely, these tumors may develop outside the genital tract, including in the mediastinum, gastrointestinal tract, pancreas, and adrenal gland (12–15). Previous studies have indicated that the incidence of uterine ATs is approximately 1% (1). However, the actual incidence may be higher because these tumors are often overlooked during sampling due to their small size and resemblance to leiomyomas (3, 16). Most uterine ATs are located in the myometrium or subserosa, particularly near the uterine horns; this is closely related to the histological finding that adenomyomas originate from the endometrial mesothelial tissue (17). The tumor locations reported in the present case series were in line with those reported previously. Nearly all uterine ATs are solitary. In the present study, all patients had solitary tumors except for one patient who had two lesions. ATs can occur at any age but are more prevalent during the reproductive age. The median age of patients in this study was 51 years, which differs from literature reports, possibly due to the small sample size. Most patients were asymptomatic, while a small number of patients presented with lower abdominal pain, menstrual irregularities, and irregular vaginal bleeding. In many cases, surgery was performed after uterine fibroids or other lesions were detected during routine physical examinations. Patients with ATs often had other gynecological conditions. In this study, uterine fibroids were the most common comorbid condition (8 cases, 50%). However, the clinical symptoms observed in these patients do not appear to be related to uterine ATs but are caused by accompanying gynecologic disorders. The disease progresses slowly, and the course varies. This tumor is almost always benign, with a favorable prognosis. Simple tumor resection is the first-line treatment. Follow-up data from previously reported cases and those from our patients revealed no local recurrence or distant metastasis after surgery.

Uterine ATs can be categorized into nodular and diffuse types, with the nodular type being more prevalent (18, 19). They are typically oval, vary widely in size, and have distinct borders, consistent with the findings of our study. Histopathologically, they are classified into glandular, angiomatous, solid, and cystic types. Typically, two or more patterns coexist, with one type predominating. The glandular and angiomatous types are the most common, whereas the cystic type is the least common. Regardless of the type or tumor size, cystic cavities of varying sizes may be observed (3, 17). Based on a comprehensive review of the literature and findings from this case series, the MRI manifestations of uterine ATs can be roughly classified into the following three types: 1) Solid type: This is the most prevalent type. Previous studies have demonstrated that solid tumors exhibit isointense signals on T1WI and low signals on T2WI. These imaging features closely resemble those of uterine fibroids, making differentiation challenging (5, 9). However, Mayumi et al. reported that solid tumors exhibited heterogeneous signal intensity on T2WI, and four lesions presented the “target sign”, defined as central hypointensity surrounded by a peripheral hyperintense annular rim (4). This finding is consistent with the pathological findings that tubular tumor cells exhibited an organized, nodular, concentrically arranged, targetoid growth pattern in 22% of cases. In addition to the T2WI imaging patterns described above, five masses in our study contained small cystic components. This MRI appearance may be closely associated with enlarged glandular spaces surrounding fascicles of smooth muscle (20). According to Akihiko et al. (5), a small number of vascular flow-void signals were observed at the margins of some masses; however, none of the cases in the present study demonstrated this feature. After contrast-enhanced scanning, the solid components showed moderate to marked enhancement, which may be related to the number of vascular spaces within the tumor cells and the interstitial blood vessels. 2) Cystic-solid type: This type is characterized by a solid component that exhibits isointense signal on T1WI and hypointense signal on T2WI, whereas the cystic component appears hypointense on T1WI and hyperintense on T2WI. The cystic areas are irregularly distributed and may be located centrally or peripherally. The corresponding pathological manifestation is that hypertrophic smooth muscle cells and fibrous septa form the basic framework of the tumor, within which glandular spaces of varying sizes and irregular distribution are interspersed. The spaces are lined by mesothelial and eosinophilic cells that primarily secrete serous fluid, providing the pathological basis for fluid signals observed within the tumor. In the present study, only two cystic-solid tumors were reported, which is consistent with previous reports. 3) Cystic type: In this study, two cystic-type lesions were reported. On MRI, these lesions appeared as cystic cavities with septa, showing low signal intensity on T1WI and high signal intensity on T2WI, consistent with fluid content. The cysts were surrounded by a thick-walled structure. After contrast-enhanced scanning, delayed enhancement of the septa was detected, consistent with prior reports (7, 10). These MRI finding may be characteristic of cystic ATs; however, because this subtype is rare, further studies are needed to confirm these findings. These imaging features may be attributable to the tumor margin being mainly composed of smooth muscle bundles, forming a thick-walled edge, whereas the central portion was filled with serous fluid and contained a few enhancing septa composed of fibrous stroma and smooth muscle bundles, consistent with pathological findings. As reported by Mitsumori et al. (5), the MRI findings of uterine ATs are closely associated with their pathological and histological features. The imaging manifestations can vary from solid to cystic, depending on the composition ratio of glandular spaces, smooth muscle, and fibrous tissue. Differences in these components directly determine the distinct signal characteristics observed on T1WI, T2WI, and contrast-enhanced scans.

In addition, Among the 15 masses (13 solid and 2 cystic-solid) in this series, the regions with hypointense signal on T2WI exhibited hyperintensity on DWI and mild hypointensity on ADC maps. The pathological specimen showed abundant tubules mixed with smooth muscle hypertrophy. This histological feature might cause water diffusion restriction. Among the four solid lesions showing a “target sign” on T2WI, no definite diffusion restriction was observed in the peripheral rim, which was considered attributable to the T2 shine-through effect rather than true diffusion restriction. Additionally, some lesions demonstrated mild hypointensity on both DWI and ADC maps. Pathological examination revealed small tubules scattered within areas of dense smooth muscle hyperplasia. This finding is consistent with the findings of Takeuchi et al. (4). These findings suggest that when focal diffusion restriction is observed in a uterine AT, a comprehensive assessment should be made together with pathological results. However, although ADC values were measured in this study, quantitative analysis was not performed, because the small sample size inherent to this retrospective study, together with the use of MRI scanners of different models and field strengths, may have compromised the accuracy of the ADC measurements. Functional imaging studies of uterine ATs are currently limited, warranting further investigation.

Because most uterine ATs arise in the subserosal region of the uterine horns, they should first be distinguished from non-uterine lesions, particularly broad ligament fibroids and tumors or tumor-like lesions of ovarian or fallopian tube origin (21). Moreover, different types of uterine ATs also require differentiation from other condition: (1) Solid type should be differentiated from uterine fibroids, because both show similar isointense and hypointense signals on T1WI and T2WI, with similar degrees of enhancement. It is challenging to differentiate them based on plain and contrast-enhanced MRI alone. Some studies have reported that on diffusion-weighted imaging, uterine fibroids exhibit a “T2 blackout” effect, characterized by low signal intensity on both DWI and ADC maps (22); this may help with identification. (2) Cystic-solid type should be differentiated from focal adenomyosis and atypical uterine fibroids. When uterine fibroids show atypical manifestations such as edema and degeneration, the fibroids are usually large (>5 cm), with heterogeneous signal intensity on T2WI and scattered or diffuse high-signal areas. In contrast, uterine ATs are generally small, and cystic changes can occur even in small lesions (23–25). If MRI shows an enlarged uterus with asymmetric myometrial thickening with cystic structures or hemorrhagic foci within the myometrium, the diagnosis of adenomyosis is supported (26–28). (3) Cystic type should be differentiated from congenital uterine cysts and peritoneal inclusion cysts. Typically, linear septa are present within cystic uterine ATs, whereas congenital uterine cysts exhibit uniform internal signal intensity without septa and are frequently associated with genitourinary developmental abnormalities. The lack of enhancement on contrast-enhanced scans is a differentiating feature (29). Among patients with peritoneal inclusion cysts, 70.6% have a history of prior peritoneal injury. This clinical feature serves as a factor for differentiating these lesions from cystic uterine ATs (30, 31).

This study has several limitations. First, it was a retrospective study with a relatively small sample size. Second, MRI examinations were performed using different scanners, introducing an unavoidable degree of subjectivity in the evaluation of imaging features. Furthermore, functional imaging assessment was limited to diffusion-weighted imaging, and quantitative analysis of ADC values was not performed. Third, because the MRI scanning plane did not align with the orientation of histopathologic sections, and pathologic evaluation relied on localized sampling rather than whole-mount large sections, a precise one-to-one correlation between imaging and histopathologic findings could not be established.

In conclusion, uterine ATs are rare and prone to misdiagnosis. Most present as solid masses. When a uterine mass demonstrates small internal cystic components or a “target sign” on T2WI, AT should be included in the differential diagnosis. Nevertheless, definitive diagnosis still relies on pathological examination. In clinical practice, it is essential to improve the understanding of cystic-solid and cystic types to minimize misdiagnosis. Moreover, the correlation between the imaging classification of uterine ATs, their pathological characteristics, and prognosis of uterine ATs require further analysis.

## Data Availability

The datasets presented in this study can be found in online repositories. The names of the repository/repositories and accession number(s) can be found in the article/**Supplementary Material**.
