# Supplementary material for: Adenomatoid tumors of the uterus: magnetic resonance imaging features with clinical and histopathologic correlation
Source: Front Oncol. 2026 Sep 8;16:1942362. doi: 10.3389/fonc.2026.1942362 (PMC13593419; doi:10.3389/fonc.2026.1942362)
Supplement: Supplementary file 1 [file SupplementaryFile1.docx]

Supplementary Material

# 1 Supplementary Figures and Tables

## 1.1 Supplementary Figures

**Supplementary Figure 6.** Same patient as in Figure 1 in the article; (A) The solid components of the tumor shows heterogeneously hyperintense on axial DWI with correspondingly mild hypointensity on the ADC map (arrow).

**Supplementary Figure 7.** Same patient as in Figure 2 in the article; The solid components of the tumor shows heterogeneously hyperintense on axial DWI with correspondingly mild hypointensity on the ADC map (arrow), In contrast, endometrial carcinoma shows marked hyperintensity on DWI with correspondingly marked hypointensity on ADC map (asterisk).

**Supplementary Figure 8.** Same patient as in Figure 4 in the article; The solid components of the tumor shows mild hypointensity on both DWI and ADC map (arrow).
