# Supplementary material for: Adenomatoid tumors of the uterus: magnetic resonance imaging features with clinical and histopathologic correlation
Source: Front Oncol. 2026 Sep 8;16:1942362. doi: 10.3389/fonc.2026.1942362 (PMC13593419; doi:10.3389/fonc.2026.1942362)
Supplement: Supplementary file 2 [file SupplementaryFile2.docx]

Flowchart of Patient Selection

pathology database search;

(Time range: between June 2016 and January 2026; Key words: adenomatoid tumor, uterus.)

patients with a histopathologic diagnosis of uterine AT (n = 71)

picture archiving and communication system (PACS) search

Excluded: patients without contrast-enhanced pelvic MRI (n = 38)

patients with a histopathologic diagnosis of uterine AT who had contrast-enhanced pelvic MRI (n = 33)

screening via PACS

Excluded:

(1) coexisting masses preventing accurate localization of the adenomatoid tumor; (n=9)

(2) poor MRI image quality or tumors measuring <5 mm in diameter. (n=6)

eligible patients (n=18)

electronic medical record review

Excluded:

(1) prior treatment (pharmacotherapy, intervention, or surgery) (n=1)

(2) incomplete clinical data (n=1)

finally included patients

16 patients, 17 lesions
