# Supplementary material for: Adenomatoid tumors of the uterus: magnetic resonance imaging features with clinical and histopathologic correlation
Source: Front Oncol. 2026 Sep 8;16:1942362. doi: 10.3389/fonc.2026.1942362 (PMC13593419; doi:10.3389/fonc.2026.1942362)
Supplement: Supplementary file 3 [file SupplementaryFile4.docx]

Table 1. MRI features of ATs

|  | age | number | location | Size (mm) | shape | margin | composition | tumor signal characteristics | | | | contrast enhancement | associated uterine leiomyoma |  |
| --- | --- | --- | --- | --- | --- | --- | --- | --- | --- | --- | --- | --- | --- | --- |
|  |  |  |  |  |  |  |  | T1WI | T2WI | DWI | ADC |  |  | |
| 1 | 56 | solitary | subserosal | 31 | oval | well-defined | solid | iso | homogeneously hypo | Mild hypo | Mild hypo | marked enhancement | + | |
| 2 | 54 | solitary | subserosal | 25 | oval | well-defined | cystic | hypo | markedly hyper, internal septa | N.A. |  | ring - like rim enhancement, internal septa with progressive enhancement | - | |
| 3 | 27 | solitary | subserosal | 25 | oval | well-defined | cystic-solid | solid component: iso  cystic component: hypo | solid component: hypo  cystic component: markedly hyper | solid component: Mild hypo | solid component: Mild hypo | solid component: moderate enhancement; cystic component: none | - | |
| 4 | 58 | solitary | subserosal | 9 | oval | ill-defined | solid | iso | Heterogeneously hypo | Mild hyper | Mild hypo | marked enhancement | - | |
| 5 | 51 | solitary | subserosal | 19 | oval | well-defined | solid | iso | homogeneously hypo | Mild hypo | Mild hypo | marked enhancement | + | |
| 6 | 49 | Multiple（two les ions） | Subserosal (right) | 65 | oval | well-defined | cystic-solid | solid component: iso cystic component: hypo | solid component: hypo cystic component: markedly hyper | solid component: Mild hyper | solid component: Mild hypo | Solid component: heterogeneously moderate enhancement; cystic component: none | - | |
|  |  |  | Subserosal (left) | 30 | oval | well-defined | cystic | hypo | markedly hyper, internal septa | N.A. |  | ring - like rim enhancement, internal septa with progressive enhancement |  | |
| 7 | 56 | solitary | subserosal | 29 | oval | well-defined | solid | iso | Heterogeneously hypo | Mild hyper | Mild hypo | marked enhancement | - | |
| 8 | 43 | solitary | intramural | 28 | oval | well-defined | solid | iso | Center: Hypo / Rim: Hyper (target sign) | Mild hyper | Center: mild Hypo / Rim: Hyper | marked enhancement | + | |
| 9 | 51 | solitary | intramural | 13 | oval | well-defined | solid | iso | Center: Hypo / Rim: Hyper (target sign) | Mild hyper | Center: mild Hypo / Rim: Hyper | marked enhancement | - | |
| 10 | 63 | solitary | intramural | 12 | oval | well-defined | solid | iso | homogeneously hypo | Mild hypo | Mild hypo | moderate enhancement | - | |
| 11 | 49 | solitary | intramural | 45 | oval | ill-defined | solid | iso | Heterogeneously hypo | Mild hyper | Mild hypo | marked enhancement | + | |
| 12 | 46 | solitary | intramural | 39 | oval | well-defined | solid | iso | homogeneously hypo | Mild hyper | Mild hypo | marked enhancement | + | |
| 13 | 51 | solitary | subserosal | 16 | oval | well-defined | solid | iso | Center: Hypo / Rim: Hyper (target sign) | Mild hyper | Center: mild Hypo / Rim: Hyper | marked enhancement | + | |
| 14 | 49 | solitary | subserosal | 26 | oval | well-defined | solid | iso | Center: Hypo / Rim: Hyper (target sign) | Mild hyper | Center: mild Hypo / Rim: Hyper | marked enhancement | - | |
| 15 | 47 | solitary | intramural | 41 | oval | ill-defined | solid | iso | Heterogeneously hypo | Mild hyper | Mild hypo | marked enhancement | + | |
| 16 | 51 | solitary | intramural | 30 | oval | ill-defined | solid | iso | Heterogeneously hypo | Mild hyper | Mild hypo | marked enhancement | + | |

Footnote: iso = isointense; hypo = hypointense; hyper = hyperintense; N.A. = Not Available; + = present; − = absent

Table 2. MRI features of solid ATs (n=13)

| imaging features | number, n (%) |
| --- | --- |
| location  intramural  subserosal  submucosal | 7(53.8)  6(46.2)  0(0.0) |
| shape (regular) | 13(100) |
| margin  well-defined  ill-defined | 9(69.2)  4(30.8) |
| capsule | 0(0.0) |
| T2WI signal features  homogeneous hypointense signal  heterogeneous hypointense signal with cystic degeneration  target sign | 4(30.8)  5(38.5)  4(30.8) |
| associated hemorrhage | 0(0.0) |
| contrast enhancement  mild  moderate  marked | 0(0.0)  1(7.7)  12(92.3) |
| associated uterine leiomyoma | 8(61.5) |
